# Effect of balance training with Biodex Balance System to improve balance in patients with diabetic neuropathy: A quasi experimental study

**DOI:** 10.12669/pjms.37.2.2336

**Published:** 2021

**Authors:** Syeda Afsheen Hasnain Daud, Mujeeb Ur Rahman, Aatik Arsh, Muhammad Junaid

**Affiliations:** 1 Syeda Afsheen Hasnain Daud, DPT, MSPT. Institute of Physical Medicine and Rehabilitation, Khyber Medical University, Peshawar, Pakistan; 2 Mujeeb Ur Rahman, BSPT, MSPT. Institute of Physical Medicine and Rehabilitation, Khyber Medical University, Peshawar, Pakistan; 3 Aatik Arsh, DPT, MSPT. Institute of Physical Medicine and Rehabilitation, Khyber Medical University, Peshawar, Pakistan; 4 Muhammad Junaid, DPT. Institute of Physical Medicine and Rehabilitation, Khyber Medical University, Peshawar, Pakistan

**Keywords:** Balance, Diabetes, Exercises, Neuropathy, Rehabilitation

## Abstract

**Objective:**

To determine the effect of balance training with Biodex Balance System in improving balance function in patients with diabetic neuropathy.

**Methods:**

A quasi experimental study was conducted at physiotherapy department of Rehman Medical Institute Peshawar from January to June 2018. Thirty-eight Diabetes Mellitus Type-II patients with diagnosis of diabetic neuropathy participated in the study. Those patients were included who were able to stand independently and who scored moderate or severe neuropathy on Toronto Scale. Participants received 12 sessions (two sessions per week) of balance training on Biodex stability system in six weeks. Berg Balance Scale and Time Up and Go test were used to collect pre and post treatment data. Paired sample T-test was used to compare pre and post treatment data because data was normally distributed.

**Results:**

The mean age of the participants was 63.08 ± 3.3 years. Pretreatment Berg Balance Scale score was 42.68 ± 3.7 which improved to 48.89 ±3.7 after the treatment (P<0.001). Similarly, pretreatment Time Up and Go test score was 17.47 ± 2.0 while post treatment score was 13.57 ±1.5 (P<0.001).

**Conclusion:**

Balance training with Biodex Balance System can significantly improve balance function in Diabetes Mellitus Type-II patients with diabetic neuropathy.

## INTRODUCTION

Diabetes Mellitus is one of the commonest non-communicable disease, affecting 246 million people worldwide.^1^ In Pakistan the estimated prevalence of Diabetes mellitus (DM) Type-II was 5,217,000 in 2000 and expected to be 13,853,000 in 2030, whereas according to WHO, Pakistan has been ranked 7^th^ in terms of DM prevalence affecting more than 6.9 million of the population and this number is going to be around 11.5 million by the year 2025.^1^,^2^ The prevalence of DM in Pakistan was reported to be 7.6% to 11%. The urban and rural prevalence of DM in Pakistan was reported to be 22.04% and 17.15%, respectively.^3^

DM and its consequences cause an array of complications. Retinopathy, nephropathy and neuropathy are the common secondary complications of DM.^4^ These secondary complications of DM are life threatening and are associated with incredible costs and human sufferings. About one third of chronic DM patients with uncontrolled diabetes develop diabetic neuropathy.^5^ The exact burden of diabetic neuropathy in Pakistan is unknown, however, there are reports that 7.5% patients at the time of diagnosis of DM had diabetic neuropathy as well.^6^ Previous studies reported that diabetic neuropathy leads to balance impairment, gait abnormalities and postural sway.^7^ In clinical settings, postural instability and balance disorders are common findings in diabetic neuropathy patients. Literature suggests that reduction in proprioception and enhanced reflex reaction time are the main causes of poor balance function in diabetic neuropathy patients.^8^ Balance dysfunction due to neuropathy adversely affects the quality of life and activities of daily life of DM patients.^9^

A number of rehabilitation interventions are applied in clinical practice to improve balance function in patients with diabetic neuropathy and many studies reported effectiveness of these interventions. Ahmad et al. reported that balance training improve static and dynamic balance in diabetic neuropathy patients.^10^ A study conducted by Akbari et al. also concluded that balance training and exercises have shown improvement in diabetic neuropathy patients who had balance and postural instability.^11^ Biodex balance system is a relatively new instrument that can be used for the assessment and management of balance dysfunction. Preliminary studies reported positive effects of balance training on Biodex balance system for the improvement of balance function, however there is limited literature available regarding its effectiveness in patients with diabetic neuropathy.^12^,^13^ Therefore there was a dire need to conducted current study in order to determine effect of balance training with Biodex Balance System in improving balance function in patients with diabetic neuropathy.

## METHODS

A quasi experimental study was conducted at physiotherapy department of Rehman Medical Institute Peshawar from January to June 2018. Ethical approval was obtained from ethics committee of Khyber Medical University (DIR/KMU-EB/EB/000489). Thirty-eight DM Type-II patients, aged 50-75 years, with diagnosis of diabetic neuropathy participated in the study. Those patients were included who were able to stand independently and who scored moderate or severe neuropathy on Toronto Scale. Those patients were excluded who had vestibular complaints, complaining of dizziness, vertigo, concomitant foot ulcers, balance impairments due to other neurological conditions such as stroke, myelopathy and cerebral ataxia and any musculoskeletal deformity i.e. amputation, scoliosis, severe degenerative joint diseases preventing participation in the study.

Purpose and details of the study were explained to the patients through information sheet and their consent was taken. Participants received balance training on Biodex stability system (Biodex 945-302, Biodex Medical Systems Inc., USA). There were two sessions per week for a duration of six weeks. On Biodex, participants were instructed to stand on a podium and stabilize themselves to keep the cursor in the middle of co-centered circle on the displayed screen/monitor. Stability level of platform was set on eight for first two sessions and then reduced to one level on every two sessions so that in the 9^th^ and 10^th^ session the stability level was four. From session four onwards, the participants performed the limit of stability exercises with Biodex Balance System. The participants performed this exercise protocol two times in each session. Total 12 sessions were given in time period of six weeks.

Berg Balance Scale and Time Up and Go test were used to collect pre and post treatment data. Higher mean score of Berg Balance Scale shows good balance function. On the other hand, lower scores of Time Up and Go test shows good balance function and higher score shows poor balance function. SPSS version 20 was used to analyze the data. Shapiro-wilk test was applied to check normality of data. Paired sample T-test was used to compare pre and post treatment data because data was normally distributed.

## RESULTS

The mean age of the participants was 63.08 ± 3.3 years. Out of 38 patients, 20 (52.6%) were males and 18 (47.4%) were females. The mean value for the Toronto scale analysis is 10. 58, which indicates that majority of the patients had moderate neuropathy.

Pretreatment Berg Balance Scale score was 42.68 ± 3.7 which improved to 48.89 ±3.7 after the treatment (P<0.001). Similarly, pretreatment Time Up and Go test score was 17.47 ±2.0 while post treatment score was 13.57 ±1.5 (P<0.001) (Table-I).

**Table-I:** Pre and post treatment scores of Berg Balance Scale and Time Up and Go test.

*Variables*	*Pre-treatment mean±SD*	*Post-treatment Mean ± SD*	*P-value*	*Mean difference*
Time Up and Go test	17.47 ± 2.0	13.57 ±1.5	0.000	-3.89
Berg Balance Scale	42.68±3.7	48.89 ±3.7	0.000	6.21

## DISCUSSION

Balance impairments are common in patients with diabetic neuropathy which negatively affects their lives.^14^ Results of current study showed that balance training with Biodex stability system can effectively improve balance function in diabetic neuropathy patients. Biodex stability system puts emphasis on the somatosensory system, thus enhancing information regarding balance. Furthermore, the visual biofeedback system of Biodex device also plays an integral role in the improvement of balance, as it provides more sensory information regarding sole pressure.^15^,^16^

The findings of current study are comparable to the studies previously conducted regarding the management of diabetic neuropathy in terms of balance training, using biodex device. Bina Eftekhar et al. reported that balance training with Biodex stability system improve mobility and balance in patients with diabetic neuropathy patients.^12^ Similarly, Akbari et al. reported that overall stability index and anterio-posterior stability index can be enhanced with balance training.^11^ Balance training with Biodex stability system not only improves balance in diabetic neuropathy patients, but it can improve balance dysfunction associated with normal aging. Siddiqui et al. showed significant improvement in the management of falls in healthy older population when balance trained on biodex stability system.^13^

It is pertinent to mention that balance training including general balance exercises and exercises in wobble board etc. can also improve balance function. Quite a few studies have reported that general physical therapy and balance training are effective in managing balance disorders.^17^-^18^ Salsabili et al. reported improvement in standing balance in DN after Dynamic stability training.^19^ Allet et al also reported effectiveness of focused/structured exercise regimen along with the balance training in diabetic patients.^20^ Morrison et al. showed decreased risk of falls in diabetic patients after balance exercise training.^21^ In a systematic review, Lesinski et al. also reported the effectiveness of balance training in healthy older adults.^22^ Elgoharay et al. showed the effectiveness of the biodex balance training in terms of improvement in balance performance and motor activities in children with cerebral palsy.^23^

Current study evaluated the efficacy of a relatively new approach of using Biodex stability system for managing balance dysfunction in diabetic neuropathy patients. There are limited studies conducted to show the effectiveness of Biodex balance training, specifically in patients with diabetic neuropathy. However, the available data suggests its effectiveness in the improvement of balance performance in patients with diabetic neuropathy but this does not suggest that the improvement in balance and postural stability with biodex balance training will also be associated with changes in patient’s confidence in performing daily life activities. The strength of this study is that it is one of the preliminary studies conducted in Pakistan which evaluated effectiveness of Biodex stability system.

### Limitations of the study

First of all, due to small sample size, generalizability of results of current study are questionable. Secondly, due to quasi experimental design of the study, it was not possible to control confounding variables. Moreover, long term effects of the intervention were not evaluated. Randomized controlled trials with larger sample size, longer follows ups and outcomes assessed on other variables are needed, to assess the effectiveness of Biodex Balance training in terms of balance performance, risk of falls and postural sway.

## CONCLUSION

From the results of current study, it can be concluded that balance training with Biodex Balance System can significantly improve balance function in DM Type-II patients with diabetic neuropathy. Randomized controlled trials, with large sample size and longer follows up are recommended to truly determine the effectiveness of Balance training with Biodex Balance System in improving balance function in diabetic neuropathy patients.

### Authors’ Contribution:

**SAHD:** Concept and study design, literature search and literature review, acquisition of data, drafting the manuscript, responsible and accountable for the accuracy or integrity of the work.

**MUR:** Concept and study design, data analysis and interpretation, drafting the manuscript, final approval of the version to be published.

**AA:** Literature search and literature review, data analysis and interpretation, critical revision.

**MJ:** Acquisition of data, Critical revision.

## References

[1] Akhtar S, Nasir JA, Abbas T, Sarwar A (2019). Diabetes in Pakistan:A systematic review and meta-analysis. Pak J Med Sci.

[2] Nation T WHO ranks Pakistan 7th on diabetes prevalence list 2009. https://nation.com.pk/15-Nov-2008/who-ranks-pakistan-7th-on-diabetes-prevalence-list.

[3] Hussain A, Ali I (2016). Diabetes mellitus in Pakistan:A major public health concern. Arch Pharma Pract.

[4] Lotfy M, Adeghate J, Kalasz H, Singh J, Adeghate E (2017). Chronic complications of diabetes mellitus:a mini review. Curr Diabetes Rev.

[5] Schreiber AK, Nones CFM, Reis RC, Chichorro JG, Cunha JM (2015). Diabetic neuropathic pain:Physiopathology and treatment. World J Diabetes.

[6] Lakhiar MA, Shahbaz NN, Bughio AH, Prakash J (2014). Frequency of peripheral neuropathy in newly diagnosed patients of diabetes mellitus iion clinical and electrophysiological basis. Pak J Neurol Sci.

[7] Alam U, Riley DR, Jugdey RS, Azmi S, Rajbhandari S, D'Aout K (2017). Diabetic neuropathy and gait:a review. Diabetes Ther.

[8] Bonnet C, Carello C, Turvey MT (2009). Diabetes and postural stability:review and hypotheses. J Mot Behav.

[9] El-Kader SM (2018). Impact of Ankle Joint Mobility on Balance Performance in Elderly Type 2 Diabetic Subjects. MOJ Gerontol Ger.

[10] Ahmad I, Hussain Dm, Singla D, Verma S, Ali K (2017). Balance Training in Diabetic Peripheral Neuropathy:A Narrative Review. JSM Diabetol Manag.

[11] Akbari M, Jafari H, Moshashaee A, Forugh B (2012). Do diabetic neuropathy patients benefit from balance training?. J Rehabil Res Dev.

[12] Eftekhar-Sadat B, Azizi R, Aliasgharzadeh A, Toopchizadeh V, Ghojazadeh M (2015). Effect of balance training with Biodex Stability System on balance in diabetic neuropathy. Ther Adv Endocrinol Metab.

[13] Siddiqi FA, Masood T (2018). Training on Biodex balance system improves balance and mobility in the elderly. J Pak Med Assoc.

[14] Timar B, Timar R, Gata L, Oancea C, Levai C, Lungeanu D (2016). The impact of diabetic neuropathy on balance and on the risk of falls in patients with type 2 diabetes mellitus:a cross-sectional study. PLoS One.

[15] Ibrahim MS, Mattar AG, Elhafez SM (2016). Efficacy of virtual reality-based balance training versus the Biodex balance system training on the body balance of adults. J Phys Ther Sci.

[16] Gehan M, Ahmed RMT, Hanan A, Amer Waleed T, Mansour Hanan H (2017). Battesha. Influence of Visual Feedback Training on Distribution of Foot Pressure in Diabetic Neuropathy Patients. Int J Diabetes Res.

[17] Grewal GS, Schwenk M, Lee-Eng J, Parvaneh S, Bharara M, Menzies RA (2015). Sensor-based interactive balance training with visual joint movement feedback for improving postural stability in diabetics with peripheral neuropathy:a randomized controlled trial. Gerontology.

[18] Pan X, Bai JJ (2014). Balance training in the intervention of fall risk in elderly with diabetic peripheral neuropathy:A review. Int J Nurs Sci.

[19] Salsabili H BF, Forogh B, Rajabali S (2011). Dynamic stability training improves standing balance control in neuropathic patients with type 2 diabetes. J Rehabil Res Dev.

[20] Allet L, Armand S, de Bie RA, Golay A, Monnin D, Aminian K (2010). The gait and balance of patients with diabetes can be improved:a randomised controlled trial. Diabetologia.

[21] Morrison S, Colberg SR, Mariano M, Parson HK, Vinik AI (2010). Balance Training Reduces Falls Risk in Older Individuals With Type 2 Diabetes. Diabetes Care.

[22] Lesinski M, Hortobagyi T, Muehlbauer T, Gollhofer A, Granacher U (2015). Effects of Balance Training on Balance Performance in Healthy Older Adults:A Systematic Review and Meta-analysis. Sports Med.

[23] El-gohary TM, Emara HA, Al-Shenqiti A, Hegazy FA (2017). Biodex balance training versus conventional balance training for children with spastic diplegia. J Taibah Univ Med Sci.

